# Supplementary material for: Complex‐centric proteome profiling by SEC‐SWATH‐MS
Source: Mol Syst Biol. 2019 Jan 14;15(1):e8438. doi: 10.15252/msb.20188438 (PMC6346213; doi:10.15252/msb.20188438)
Supplement: Supplementary file 8 — Dataset EV7 [file MSB-15-e8438-s008.zip › feature_plots_string/Q9H0I9.pdf]

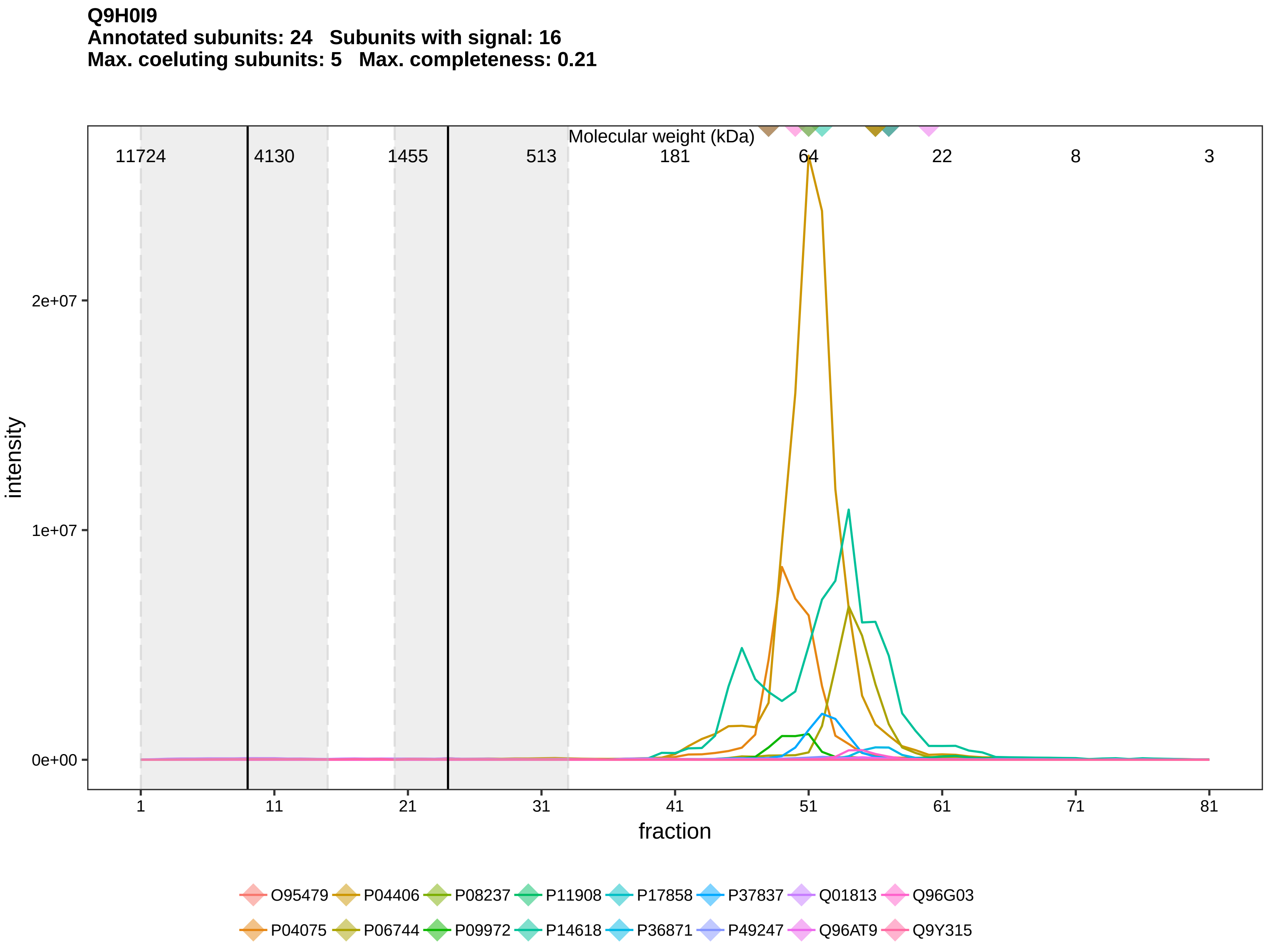

Q9H0I9
Annotated subunits: 24 Subunits with signal: 16
Max. coeluting subunits: 5 Max. completeness: 0.21
Molecular weight (kDa)
11724
4130
1455
513
181
64
22
8
3
intensity
2e+07
1e+07
0e+00
1
11
21
31
41
51
61
71
81
fraction
O95479
P04406
P08237
P11908
P17858
P37837
Q01813
Q96G03
P04075
P06744
P09972
P14618
P36871
P49247
Q96AT9
Q9Y315
